# Challenges in the veterinary microbiology diagnostic laboratory: a novel *Acinetobacter* species as presumptive cause for feline unilateral conjunctivitis

**DOI:** 10.1099/acmi.0.000118

**Published:** 2020-03-20

**Authors:** Ana P. Vale, Bernadette Leggett, David Smyth, Finola Leonard

**Affiliations:** ^1^​ School of Veterinary Medicine, University College Dublin, Dublin, Ireland; ^2^​ Brookpark Veterinary Clinic, Dunmanway, Co. Cork, Ireland; ^†^​ Present address: Institute of Technology Sligo, Sligo, Ireland

**Keywords:** Veterinary microbiology, microbiology diagnostic laboratory, *Acinetobacter*, antimicrobial resistance

## Abstract

The present study highlights challenges in the veterinary microbiology diagnostic laboratory in the identification of bacteria responsible for infections in veterinary settings, particularly when evidence-based data is lacking. A 1.8-year-old neutered male domestic cat (FIV/FeLV negative) was presented to a veterinary practice in April 2016 with a history of left unilateral mild conjunctivitis that was empirically treated with fusidic acid and chloramphenicol. In January 2017, the same animal was presented with chronic left unilateral conjunctivitis and an eye swab was submitted for microbiological culture and susceptibility testing. Significant growth was not detected in two samples tested. Finally, in February 2017 another eye swab produced a slow growing pure culture identified by VITEK 2 as *Neisseria cinerea* (94 % confidence). Given the morphology and multidrug resistance profile of the isolate a 16S rRNA PCR was performed for definitive identification. The nucleotide sequence of the PCR amplicon was 99 % homologous to *Acinetobacter equi* sp. nov. strain 114. Veterinary microbiology diagnostic laboratories play an important role worldwide, not only in preserving animal health and welfare but also in controlling the spread of zoonotic pathogens. The lack of evidence-based information on the ocular microbiome of healthy cats and the complexity of bacterial ecosystems renders the interpretation of results difficult. A further problem for both the laboratory and the clinician is the lack of interpretive criteria for antibiotic susceptibility test results for some types of infections in animals (including those caused by *Acinetobacter *) and the complete unavailability of criteria for topical antibiotic preparations.

## Background

In everyday practice, veterinarians are frequently presented with suspected bacterial infections in their patients, which are medicated with antibiotics. Despite the antimicrobial resistance (AMR) problem threatening human and animal health, veterinarians often fail to request microbiological diagnostic tests and decide instead to approach the treatment of infections empirically, with the objective of improving patient wellbeing and increasing client compliance. Furthermore, even when clinical samples are collected and laboratory testing is carried out, unusual growth requirements of organisms, lack of evidence-based information, unavailability of standardized procedures or other difficulties may challenge the ability of diagnostic laboratories to provide accurate results.

This study highlights how conventional methods used in the diagnostic laboratory can struggle to correctly identify bacteria potentially causing infections and the need for considering uncommon multidrug-resistant bacteria as possible causes of infections in companion animals, especially when evidence-based data is lacking.

## Case presentation

A 1.8-year-old neutered male domestic shorthaired cat (FIV/FeLV negative) was presented to a veterinary practice in April 2016 with a history of left unilateral mild conjunctivitis.

The cat was discharged with fusidic acid (Isathal) 10 mg g^−1^ eye drops (bid) for a week. In July 2016, the animal returned with no signs of improvement. A fluorescein eye stain tested negative and the Schirmer tear test result was 9 mm. Chloramphenicol (Chloromycetin) 0.5 % w/v eye drops were prescribed (qid) for a week. In January 2017, the same animal was presented again with chronic left unilateral conjunctivitis still unresolved. The fluorescein eye stain tested negative again and the clinician decided to collect an eye swab to submit for microbiological culture and susceptibility testing in a local veterinary diagnostic laboratory. No significant growth was detected. Following this result a second swab was collected from the affected eye and sent to the University College Dublin (UCD) Veterinary Hospital microbiology diagnostic laboratory. As before, significant isolates were not recovered during this investigation. Information on further treatment at this stage is not available.

At the end of February 2017, the clinical signs had not resolved and thus another eye swab was collected and sent to the UCD Veterinary Hospital microbiology diagnostic laboratory. Pending results, eye drops containing 1 mg dexamethasone, 6000 IU polymyxin B sulphate, 3500 IU neomycin sulphate 0.1 % w/v (Maxitrol) were prescribed (tid) for 7 days. Slow-growing colonies that were identified by VITEK 2 (Biomerieux) of *Neisseria cinerea* were recovered on blood agar and a Kirby–Bauer disk susceptibility test was performed. Marbofloxacin 100 mg sid PO was prescribed for 10 days based on the antimicrobial susceptibility profile reported. The condition resolved, and clinical signs of conjunctivitis were not detectable when the cat was presented again in June 2017 for vaccination.

## Investigation

After the initial unsuccessful attempts to identify significant micro-organism/s as causing the unilateral conjunctivitis, a pure culture of a Gram-negative, oxidase-negative, catalase-positive, fastidious, slow-growing bacterium was recovered on blood (BA) and chocolate agar (CA) media from the third eye swab collected.

Identification of the organism was attempted using the VITEK 2 GN ID card (Biomerieux) according to the manufacturer’s instructions. The organism was identified as *Neisseria cinerea* with 94 % confidence. Despite the challenge of growing this organism in the laboratory, antimicrobial susceptibility testing by disc diffusion assay was carried out in accordance with the CLSI guidelines. The micro-organism was tested against amoxicillin+clavulanic acid (AmCl), florfenicol (Flor), fusidic acid (Fus), gentamicin (Gent), marbofloxacin (Marb), neomycin (Neo), polymyxin (Poly), tetracycline (Tet), trimethoprim-sulphamethoxazole (TrS), cephalotin (Cep), cefovecin (Cefo), minocycline (Mino) and chloramphenicol (Chlo) and was resistant to AmCl, Fus, Tet, TrS, Cep, Cefo and Chlo (Table 1) according to interpretive criteria for Gram-negative organisms in VET01-S2 [1].

**Table 1.** Results of antimicrobial susceptibility testing by disc diffusion

Antibiotic	Susceptibility
Amoxicillin+clavulanic acid	R
Florfenicol	S
Fusidic acid	R
Gentamicin	S
Marbofloxacin	S
Neomycin	S
Polymixin	S
Tetracycline	R
Trimethoprim-sulphamethoxazole	R
Cephalothin	R
Cefovecin	R
Minocycline	S
Chloramphenicol	R

R indicates resistant and S indicates susceptible.

Given the morphology, slow growth rate, antimicrobial resistance pattern (multidrug resistance) and the persistence of clinical signs after empirical treatments, it was decided to proceed with a 16S rRNA PCR for definitive identification of the isolate. PCR was performed as described elsewhere [2] and a PCR product of approximately 1300 bp was sequenced by Sanger (Eurofins). The nucleotide sequence of the PCR amplicon was 99 % homologous to *Acinetobacter equi* sp. nov. strain 114, submitted to NCBI with the reference sequence: NZ_CP012808.1 [3].

Common infectious causes of conjunctivitis in cats include bacteria such as *Chlamydia felis* and *Mycoplasma* spp. and viruses such as feline herpesvirus type 1 (FHV-1) and feline calicivirus (FCV) [4–7].

Fungal or mycoplasma organisms were not isolated from the samples submitted to the UCD Veterinary Hospital microbiology diagnostic laboratory. PCR-based diagnostics for *Chlamydia felis* and FHV-1 were not available in this laboratory.

## Discussion

Conjunctival pathology associated with bacterial and viral agents is a common problem in small animal practice [4–8]. FHV-1 and *Chlamydia felis* frequently cause conjunctivitis in cats; published studies suggest that approximately 80 % of cats are latent carriers of FHV-1 and *Chlamydia felis* is responsible for 60 % of feline conjunctivitis [5–7]. The lack of PCR investigation for FHV-1 and *Chlamydia felis* is a limitation of the present study; however the clinical presentation including the absence of upper respiratory tract disease, the unilaterality of the problem and the patient’s medical history (age/household) supported the prioritization of other differential diagnoses.

The case reported here highlights the importance of requesting diagnostic testing to assist with selection of appropriate treatment. Additionally, it illustrates how infections may be caused by uncommon micro-organisms that standard procedures can fail to identify, specifically in conjunctivitis cases where causative agents are difficult to detect [5]. A further problem for both the laboratory and the clinician is the lack of interpretive criteria for antibiotic susceptibility test results for some types of infections in animals and the complete unavailability of criteria for topical antibiotic preparations in either humans or animals [9]. Interpretive criteria for *Acinetobacter* species are limited to human pathogens due to the poor reliability of commercial antimicrobial susceptibility testing devices and insufficient breakpoint interpretations for the antibiotics available [10]. Thus, criteria for systemic treatment of other Gram-negative infections of dogs and cats were used by the laboratory in this case. The practising veterinarian chose systemic therapy when selecting treatment on the last occasion when the cat was presented to him and it appears that the *in vitro* result of susceptibility to the fluoroquinolones was reflected by a successful treatment outcome *in vivo*.

The lack of evidence-based information on the ocular microbiome of healthy cats [11, 12] and the complexity of bacterial ecosystems renders the interpretation of ocular microbiological results difficult. Nevertheless, a strong clinical history built upon an excellent clinical examination significantly strengthens the value of microbiological diagnostics. Importantly, diagnostic microbiology laboratories provide essential information on infectious diseases and antimicrobial resistance that are emerging and spreading worldwide.

Currently, identification of bacterial pathogens relies mainly on culture and/or molecular methods, including 16S rRNA, which is a gold-standard method for bacterial identification [13, 14]. Novel tools including MALDI-TOF MS and next-generation sequencing (16 S-23S rRNA) are now available however, high costs and applicability to routine diagnostics limit their use in veterinary diagnostic laboratories [14].

Bacteria of the genus *Acinetobacter* comprise species that are ubiquitous in soil and water. Some species, including *Acinetobacter baumannii *, are opportunistic pathogens in both human [15] and veterinary settings [16]. In addition, they may cause nosocomial infections and are often multidrug resistant[17]. *Acinetobacter* spp. have been reported to cause acute/chronic conjunctivitis, corneal ulcers and dacryocystitis in humans [18, 19]. Although ocular infections with this organism in animals have not been reported, a UK study identified *Acinetobacter* spp. as the most frequently isolated bacteria from the normal conjunctiva of horses [20].

Veterinary microbiology diagnostic laboratories play an important role worldwide, not only in preserving animal health and welfare but also in controlling the spread of zoonotic pathogens, responsible for 60 % of human infections [21]. Future availability of new technologies will facilitate the correct identification of micro-organisms and their antimicrobial susceptibility profiles. Furthermore, it is essential to promote evidence-based research that can deliver relevant data to clinicians and microbiologists.
